# State of Lunacy in England

**Published:** 1857-07-01

**Authors:** 


### Art. IX.—STATE OF LUNACY IN ENGLAND.

The Annual Reports published by the authority of the Committee of Management of the various county asylums in England generally contain a valuable body of evidence relative to the condition of the pauper portion of the insane population of this country. The value of these Reports would be considerably enhanced if the resident medical officers had the space and leisure to enter more minutely into detail respecting the numerous cases of interest which must daily come under their observation and treatment. We are fully aware of the onerous and anxious duties imposed upon the medical superintendents of our large county asylums, and of the little time they have to devote to literary work. We wish it were otherwise. The evil complained of can only be obviated by the appointment of persons to relieve the medical officer of the anxious mechanical work which, according to present arrangements, occupies so much of his valuable professional avocations.

It appears from the last Report of the medical superintendent of Bedford Lunatic Asylum that a larger number of patients by 14 were received into the asylum than in the preceding year, those admitted during the year 1855 being 40 males and 57 females, and throughout the year just closed 54 males and 57 females. Of these 70 were cases of mania, 16 melancholia, 17 imbecility, 2 dementia, and 5 idiots; amongst whom the combinations of physical disease are so numerous, and many of so incurable a nature, as to reduce the prospect of recovery to a very small amount.

The discharges numbered 54, the average of recoveries being nearly equal to the increase of admissions; 8 only were discharged relieved, 3 removed by friends, and 1 transferred to another asylum.

The mortality, although very large, has been a fraction under the average (upon the whole numbers) of last year. As many as 12 have died within three months of their being brought here, 5 of whom did not survive a fortnight. One male, an epileptic, 79 years of age, and having been bed-ridden for years from contracted limbs, and nearly exhausted from the journey, died on the twelfth day. A female, aged 68, with disease of the heart, died on the fourth day from exhaustion, having been some time without rest, and refused her food previous to admission. A female in the last stage of pulmonary consumption lived but seventeen days; and one very distressing case of a female in the prime of life, whose youthful days had been spent in debauchery, but who, for eleven years, had been an industrious and faithful wife, was brought to the asylum, worn out from constant excitement, having a large wound on the leg, with ulcerations from ligatures on the wrists and ankles, sunk on the fourteenth day. The two last-mentioned patients were reported to have refused food for nearly a week, but took every kind of nourishment offered to them from the moment they were in the asylum.

Mr. Denne reports that no suicide has occurred in the asylum during the last year, notwithstanding 36 suicidal cases were admitted into the asylum. Mr. Denne and the Commissioners of Lunacy report favourably of the condition of the asylum.

The last Report of the Buckinghamshire Lunatic Asylum contains a vindication of the Committee, whose conduct has been so seriously impugned for their summary dismissal of Mr. Millar from the medical superintendence of the asylum.

We consider that Mr. Millar has published a satisfactory refutation of these charges, to which we refer our readers interested in this matter.*

It appears from the Report of the North Wales Lunatic Asylum for 1857 that—

"The inmates of the asylum under our care have been blessed with excellent health and total freedom from epidemic disease during the past year:—

| | |
|---|---|
| The admissions have been . . . . | 68 |
| The discharges:—viz., cured, 32; relieved, 15; not relieved, 11 . . . | 58 |
| The deaths . . . . . . . | 16 |

"The causes of death have been such as are common to the insane:—viz., consumption, general paralysis, debility, and exhaustion;—complaints over which medical science has little control."

The medical superintendents, Dr. R. L. Williams and Mr. George T. Jones, when referring to the mode of conveying patients to the asylum, justly observes:—

"There is nothing that a lunatic will longer recollect and more indignantly resent than being deceived. It is much better to tell a patient, calmly and candidly, that it is deemed essential to his own welfare and that of his family, that he should be sent from home for a time, and that he will be kindly treated by those who have the care of him at his temporary abode. Even the employment of gentle force, though seldom required, is preferable to deceit—the one will be forgotten and forgiven, and the other never. When the patient has been brought to the asylum by stratagem, we are always looked upon with suspicion and as participating in the fraud practised upon him;—confidence in us is lost, and our influence lessened."

When alluding to the necessity for the establishment of a

* A Refutation of the Fourth Annual Report of the Visitors of the Bucks County Lunatic Asylum. London: Renshaw.

middle-class asylum, a scheme we have often advocated in this Journal, the subjoined pertinent remarks are made :—

"The law has wisely provided for the pauper when visited by affliction in its worst form—insanity : but alas ! for the decayed tradesman, the struggling medical practitioner, weighed down by anxiety, disease, and misfortune, the deserving and ill-requited curate, and the poor ill-paid and overworked governess—one of the most frequent victims of insanity ! Probably nurtured in the lap of luxury and indulgence, misfortune or imprudence on the part of her parents reduce her to seek employment little congenial to her former habits—overworked mentally, finding little sympathy from her employers, with little or no society, and slighted even by menials, her health gives way, her mind succumbs, and insanity ensues. Often have we seen such sensitive young creatures consigned to herd with paupers in our public asylums. In our own we could unfold tales of distress and misery amongst this class, which would harrow the feelings of the reader; but delicacy forbids our entering upon details beyond a brief allusion to one or two cases, which cannot meet the eyes of themselves or their friends. In one case, a very interesting young creature, a foreigner, became violently maniacal from excessive mental labour. What would have become of the poor young lady, we cannot conjecture, had she not attracted the attention of a reverend gentleman*—an active and energetic member of our committee, and whose indefatigable zeal in all that concerns the welfare of this institution we beg thus publicly to acknowledge. This gentleman had the poor sufferer conveyed to this asylum, where she soon recovered; and she was subsequently admitted into an excellent institution in London, till she could be restored to her friends in France.

"The fate of the other sufferer was not so fortunate. She was the daughter of a once prosperous and wealthy gentleman, who fell into commercial difficulties. He died, and his family were left destitute, and his daughters were obliged to resort to teaching as a means of subsistence. Our patient soon became unequal to the labours and anxieties of a life to which her former habits were quite inadequate, and insanity was the consequence. After long and futile attempts by her friends to treat her at home, she was brought to this asylum, and admitted at the expense of her struggling relatives as a second-class patient. She had advanced considerably towards recovery, when the pecuniary difficulties of her poor friends obliged them to remove her to their own home, where she soon relapsed, and became as bad as ever; and she is now an inmate of a pauper asylum, where she will probably end her days !"

It appears from the last Report of the Essex Lunatic Asylum that—

"On the 25th December, 1855, there were 334 patients in the Asylum, namely—139 males and 195 females.

"The number of patients admitted during the year was 134, viz.—

* Rev. W. H. Owen, Senior Vicar of St. Asaph.

70 males and 64 females; and the total number of cases under treatment in the course of the year was 468; of which number only about 80 were curable—notwithstanding the number of old and incurable cases 52 were discharged recovered; 5 were removed improved, 1 unimproved, 2 escaped, and 38 died; there now remain in the House 156 males and 214 females—total 370.

"A considerable increase has taken place in the number of patients; the lowest number was 334 and the highest number 373; while the daily average number in 1855 was only 321."

It will—

"Be perceived that the number of recoveries bears a very respectable proportion to the number of curable cases under treatment."

The following extract from Dr. Campbell's valuable Report contains matter of great interest:—

"Twenty-five patients were admitted in whom a suicidal propensity formed a feature of derangement, and many of them, after admission, manifested the intention of destroying themselves; but the customary superintendence exercised over such persons has fortunately prevented any suicide. In mentioning this, I only do so to bear testimony to the fact, that every practicable care was employed to avert so distressing an event, for, notwithstanding the greatest vigilance and best management, but for the kindness of Providence, such accidents will sometimes occur. My own conviction is that to the determined suicide opportunity is seldom wanting.

"Of the various means whereby suicide is attempted by the insane, that of starvation is often persevered in with the greatest obstinacy, and in several cases, after every effort to conquer the opposition of the patient had been used without success, I was obliged to have recourse to compulsory feeding.

"To distinguish when the refusal of food arises merely from hallucination and when it is the consequence of organic lesion, requires no small degree of attention. One patient who formed the most determined resolution to destroy herself by starvation, was reduced almost to the appearance of a skeleton, and extremely enfeebled. From the healthy state of the tongue and absence of any bodily disease, there was no reason to apprehend a loss of digestive power. Nutritious fluids, with the addition of wine, were introduced twice a day into the stomach by means of a feeding instrument, and cod-liver oil was administered in the same way. She persisted in her lamentable purpose for nearly four months, when becoming convinced that she would not succeed, her resolution relaxed, and she began to take food voluntarily. From that time she rapidly improved in her bodily health, and I hope may yet be discharged well.

"In several other cases also had the means mentioned not been resorted to, the patients must have sunk and died from inanition.

"I regret to say that in some instances this treatment was imagined by patients to be the very excess of cruelty, and stated to relatives as such when they visited the institution. In the great majority of cases however, the feeling was very different. Many patients have expressed much gratitude for the humane treatment they have received, and I have during the past year been much gratified by frequent visits from patients after their discharge, and, also, by receiving several letters evincing good feeling, and gratitude for the attention they received. The following letter from a pauper patient is worthy of being noticed:—

" 'Notting Hill, London, January.

" 'Honoured Sir,—The remembrance of past mercies often comes very forcibly to my mind. When I think of all that has been done for me, I feel I am a great debtor, and now that God has prospered me so much, not one thing has failed me in all I have undertaken, but hitherto has He helped me. I think I may venture to ask this humble request, that the enclosed 5*l.* may be received as a donation to the Asylum, feeling deeply sensible I owe all my present comforts to that unerring Hand that placed me there, and to you and others that are connected with the Asylum, by being made instrumental in doing so much for me. I earnestly trust you will take it as the only little return I can make, with kind duty

" 'I am, honoured Sir, your grateful servant,

" 'Dr. Campbell." " 'E. C.'

"Respecting the causes of insanity in the patients admitted, it will be observed by the tables that hereditary predisposition forms no small number, and I regret to have occasion again to repeat that a considerable number became affected with their malady in consequence of the abuse of spirituous liquors. Some of these have been discharged recovered; and although I do not place implicit confidence in their veracity, yet I have reason to believe that the account which they give of the origin of their deplorable habit is not far from the truth. Some allege that they became addicted to the use of intoxicating liquors for the purpose of relieving bodily pain, or the depression of mind occasioned by poverty and want—some to the pernicious habit of having been indulged in early years. Some ascribe their evil propensity to bad example; and not a few females to the use of cordials administered to them remedially; and especially during in-lying, by kind but injudicious friends. The habit of drunkenness in whatsoever way it may have been induced, is too often incorrigible; and among its baneful consequences we meet with many instances not only of temporary insanity, but of permanent lunacy and imbecility of mind.

"The discipline of a Lunatic Asylum, and the dread of being again confined, have I believe in some instances induced the drunkard to observe temperance. But it is easier to prevent than to cure, and in not a few of the cases which have come under my observation, I have no doubt that the propensity to drunkenness might have been prevented. To children and to ailing or enervated adults, the use of so dangerous a foe to the health, both of the body and of the mind, as ardent spirits is allowable only when confined to immediate medical prescription; and great care should be taken to guard against the insidious approach of the enemy in disguise, whether in the inviting form of some luscious liqueur, or under the friendly aspect of stomachic tincture or cordial balm.

"The treatment of the patients has been varied according to the features and the causes of their lunacy.

"The moral management by the usual means of correcting their evil habits and propensities, and of regulating as much as possible their conduct and behaviour, has been of much utility. Internal remedies with good nourishing diet and, frequently, stimulants, by restoring the general health, have been of great service.

"The warm bath sometimes conjoined with the affusion of cold water on the head of the patient, has been used with advantage. Exercise and employment in the open air have been of general utility, and in some cases of maniacal excitement very great benefit has been derived from the use of the cold shower bath. Mechanical restraint has not been employed in any case during the year, but it has been found necessary in several cases of high excitement to have recourse to seclusion, and a reasonable degree of coercion, which cannot be altogether dispensed with in Institutions of this nature, though conducted on principles of the greatest humanity and tenderness to the patients.

"I have in former reports noticed the importance of religious services in the moral treatment of the patients, and further experience makes me feel if possible more confirmed in my opinion, of the propriety of affording patients in an Asylum the benefit of public worship.

"I have repeatedly during the past year had occasion to notice the fixed attention during service, of many patients, who, on other occasions, were particularly remarkable for wandering of thought, and unsteadiness of purpose. This circumstance is of itself a strong recommendation of the practice, evincing its tendency to favour the recovery of that control of the mental faculties, in which soundness of mind may be regarded, in a great measure, to consist. The benevolent Author of the Gospel restricts the communication of his doctrines to no particular grade of understanding. He who has but one talent, is no less favoured than he who has three.

"In regard to the amusements of the patients, I can recall nothing of any moment to which in former reports I have not alluded. The amusements of the insane poor are derived very much from their daily occupation, still a considerable number of them evince a taste for reading; consequently it is of much importance that the means should be supplied for the enjoyment of this source of relaxation, when their inclinations prompt them to seek it. Influenced by these considerations, as well as by the desire of affording the patients every comfort, a considerable number of publications in weekly and monthly parts were provided during the year, and I gladly take this opportunity of acknowledging the receipt of some very appropriate presents of books, from a lady in the neighbourhood, for the use of the patients; an example I should be glad to see generally followed. The game of cricket was introduced, and continued in high favour, a large number of the patients with some of the officers and attendants joining in it during the summer evenings. Living in a state of seclusion from the world, and shut out from the innumerable incentives to mental activity, novelty forms a prominent feature of every attempt made to sustain their mental and physical energies. Every addition consequently made to the means of affording them proper gratification, serves to extend the means of moral treatment, and innocent amusements often no less beneficially than useful employment serve to displace bewildering hallucinations. I should be glad if extended facilities were afforded for musical parties during the winter months.

"Many of the patients, and especially those in whom the malady was not in a chronic or confirmed state, derived great benefit from bodily labour. A large number have been almost daily employed at various kinds of out-door work; one has been employed regularly in the carpenter's shop, two or three in the tailor's shop, and five in the shoemaker's shop, three have been employed painting and white-washing, thus assisting to forward some necessary repairs. The females were principally employed in the washing-house, work-room, laundry or linen-room, kitchen, and in assisting the attendants, especially in the operation of cleaning. On reference to table No. 13, it will be seen that a large quantity of work has been done; in addition to which several little articles were made for my family and the other officers, affording pleasure to the patients, and in some cases attended with very beneficial results. It is often no easy task to induce patients to submit to any kind of labour. Not a few of them obstinately refuse to work, because any such employment would be degrading to persons of their imaginary high rank and unbounded wealth. Others again declare, that they will not work unless they receive regular wages for their labour. Others think they are unjustly confined, and that if their work is found profitable they will be longer detained in the Asylum. But by persuasion, example, and little indulgences, their obstinacy is at length generally subdued.

"Some patients whose cases appeared to be almost hopeless, recovered to a considerable extent by working on the farm. In one remarkable instance of recovery in which the most fanciful and gloomy anticipations predominated whenever the patient was warmed by toil, his mind was relieved. It seemed as if the vapours of the brain exhaled with the sweat of the brow. The beneficial effect of labour is on the mind two-fold: it serves not only to dissipate gloomy and incoherent thought by day, but also to prevent the distressing restlessness of the night, by preparing the patient for sound repose.

"During the year I have to encounter no small degree of trouble by the often-expressed wishes of parties to remove their insane relations, under the impression, that if they can work here, they are able to work at home. Several patients are now in the Asylum whom it might not be easy at all times to prove to be insane, and yet whose minds have long been incurably unsound, and who if liberated would be extremely troublesome to their friends, and to society. I would strongly impress upon such persons how difficult it is in many cases of real lunacy to form an opinion, or even to decide whether the patient be actually insane or not. Persons who are unacquainted with the great variety in the forms and degrees of lunacy, are apt to suppose, that to detect any aberration from soundness of mind does not require much experience, and it is not uncommon for parties to visit for the purpose of judging of the state of the malady, while in some cases no rational opinion can be formed, even by those who understand the subject, without a particular and careful retrospect of past symptoms."

The "Bethlem Hospital Report" for 1857 is replete with valuable matter. The statistics of the asylum are as follows:—

"During the past year 61 male and 110 female patients, making a total of 171, have been admitted into the curable establishment; and during the same period, in that department, 111 patients have been discharged *cured*, 78 discharged *uncured*, and 6 have *died*. Three deaths have occurred this year among the incurable female patients, after a residence respectively of 17, 31, and 33 years; also that 41 criminal lunatics have been admitted, and that 3 have died in that class, one of whom had been in the Hospital 26 years, and another 33 years. One male patient is, at the present time, absent 'on leave,' making 302 in the Hospital on the 31st of December, 1856. These for brevity may be arranged in the following Table:—

| | Males. | Females. | Total. |
|---|---|---|---|
| Curable . . . . . . . . . . | 47 | 80 | 127 |
| Incurable . . . . . . . . . | 38 | 36 | 74 |
| Criminal . . . . . . . . . | 81 | 20 | 101 |
| | 166 | 136 | 302 |

We cite the following interesting case of melancholia and delusions depending upon physical disease:—

"A. S. was admitted into Bethlehem Hospital July 7, 1856. Her previous occupation had been that of a domestic servant. She was unmarried, but the mother of two children, the youngest being two years old. The medical gentleman who had previously attended upon her, and certified to her insanity, reported, 'a strong tendency to commit suicide; a continual desire to have her inside opened; a belief that she had no bowels; and that her character and temper had lately undergone a great change, evincing hatred to those she had formerly loved and esteemed.' She was certified also 'to be dangerous to others.' On admission her physical health was feeble, pulse weak, appetite capricious, bowels constipated; mentally she appeared suffering from continued melancholia, with obstinate taciturnity. She acknowledged her despondency and attributed it to her physical state, aggravated by the neglect of the father of her children. If conversation could be forced from her it referred mainly to the state of her inside, which she considered had been misplaced, that her entrails had left her, and that she must be cut open. On these subjects she was unyielding, and became angry if any attempt was made to reason with her. The frequent constipation of her bowels, to a great extent, supported her impression that she had no inside. A report that she was suffering from a hernia induced personal examination, when a large tumour was discovered. She afterwards acknowledged her belief that into this sac her intestines had slipped. For the first fortnight after her admission the ordinary moral treatment of the Hospital was employed: a generous diet, wine, laxatives, and morphia, in grain doses at bed-time, to procure sleep. During this time no improvement in her mental symptoms was manifest. Her despondency remained unrelieved, and the delusions unchanged. Mr. Lawrence was then requested to see her; and after a careful examination of the tumour, and a full persuasion that no hernial sac existed, proposed to remove the tumour, hoping, by this measure, to cancel the delusions that appeared to depend so much upon its existence. The patient offered no objection to the operation, and on the 24th inst., her bowels having been prepared by aperient medicines, followed by an injection, Mr. Lawrence removed the tumour, which was of cellular structure, and weighed (after a large quantity of fluid had escaped) one pound and three ounces. The operation was performed by Charrière's écraseur, an instrument fully described by Mr. Spencer Wells in the 'Medical Times and Gazette' of October 11th. It had a peculiar advantage in this case, in diminishing materially the bleeding consequent upon the operation. The patient pleaded so earnestly for chloroform that it was administered, but it did not succeed in obtaining the desired insensibility. She, however, submitted to the operation with tolerable fortitude: but little blood was lost, and in a few hours she had some calm and refreshing sleep. The operation lasted about half-an-hour; the pain increased apparently with each click of the screw, by which additional pressure was produced around the point of constriction. For two or three days there was slight constitutional disturbance, a little feverishness; these symptoms soon gave way to general improvement, though mentally she retained many of her delusions, and grumbled sadly at the pain that had been given her. On the 9th of August (sixteen days after the operation) she was reported to be 'going on well, as regarded the operation, and her mental symptoms improved.' Her appetite now increased, occasionally aperient medicine was necessary; her spirits were good; and her conversation, now cheerful and unrestricted, referred only so far to her late illness as to complain of smarting annoyance from the granulating surface of the wound. To the 17th of October her mental and physical improvement continued: she became robust, happy, and industrious. Beyond aperients, the only medical treatment required was occasional sedative draughts at bed-time, and twice in the week nitrate of silver was applied to the granulations, which were a little too exuberant. On the 21st of October she returned to her family quite well.

"The points in this case which, perhaps, merit the most attention are, 'The power that the physical condition exercised over the mental state in causing the melancholia and delusions; the desirability of removing the cause before treating the effect;' and thirdly, 'The character of the instrument used by Mr. Lawrence.' As regards the latter point it may be worth while to state, that much annoyance to the patient, and difficulty to the operator, in obtaining free access to the part, was obviated by the mechanical arrangement of the instrument; that little more than an ounce of blood was lost during the operation; and that, after the removal of the tumour, although a surface of three square inches was exposed, no vessel was tied—a feature in an operation of considerable importance when the patient has shown great suicidal determination."

It appears that of—

"The 111 patients discharged *cured*, 83, or an average of 75 per cent., had not been insane more than three months at the time of their admission; whilst of the 78 discharged *uncured*, only 35 had been insane so short a time as three months, and a large majority of the remaining 43 had been insane six months and upwards."

In speaking of the criminal lunatics, Dr. Hood observes:—

"The females of this class are few in number (20) and, not differing much from each other in habits and general character, can be permitted to associate together without inconvenience. Their wards are well lighted, ventilated, and warmed. Their single airing court is as much as is required; and sufficient healthful occupation is provided for them in the care of their own wards, and in ironing, mangling, and needle-work.

"The case is very different with the males, who consist of three distinct classes, viz.:—

"1st. Men of education and refinement who, under the deep affliction of insanity, have committed the acts which have placed them in their present position.

"2nd. Men of little education, and humble position in society, who being similarly afflicted have similarly acted, but who, in general, are harmless, inoffensive, and controllable.

"3rd. Men of characters the most debased, whose associates have ever been of the worst description, and whose lengthened career of crime has been suspended by incarceration in a prison, from which they are brought to the hospital on account of insanity, which, in many instances, was only feigned.

"The first two of these classes can be united in society without inconvenience, but they regard with horror any admixture with them of the third; yet it is not right to allow that large amount of blasphemous and indecent language which is certain to result from permitting the third class to associate exclusively together—nor is such a proceeding safe. It is well known that the *insane* rarely act in combination for a common purpose; but this class consisting of individuals, many of whom are not insane, are capable of any conspiracy, and, it is believed, would gladly sacrifice the life, as they often threaten to do, of any officer or attendant if a favourable opportunity were offered them, to give a colour to that pretended insanity which they have but too successfully assumed. It is needless to remark on the amount of vigilance that is requisite in the care, and the anxiety that must accompany the superintendence, of such men, removed from the rigid discipline of a hulk or convict prison, to which has, in many cases, been added the security of solitary confinement, to associate with other convicts unshackled and unrestrained in the wards of a lunatic asylum.

"The first of these classes is little inclined to manual labour; these patients prefer intellectual pursuits, with the means for which they can be and are abundantly provided. Their exercise is, unfortunately, with the other classes in the single airing-ground appropriated to the whole.

"The second class is extensively employed in manual labour to their own gratification, and to the benefit, not only of themselves, but all the Hospital.

"To the third class as much occupation is afforded as circumstances will admit, but, in general, they are not to be trusted.

"The wards for these patients present an unsatisfactory contrast with the rest of the establishment (and produce an unfavourable impression on all visitors). Yet it is not, perhaps, judicious under present circumstances to recommend much alteration in them. They are obviously unsuitable for the three classes now occupying them. In proof of this it is only necessary to advert again to the fact that there is only one airing-court for all."

The statistical records of the Hanwell Asylum for 1857 are as follows:—

"The total number of patients, of all descriptions, received into the asylum in the past year, was 80 males and 60 females, including therein 6 cases of re-admission. There have been discharged cured, 25 males and 22 females; 37 males and 35 females have died, as has been already stated; 13 males and 4 females have been removed to other asylums, or discharged into the hands of their friends; and there remained in the asylum at the close of the year, 439 males and 584 females."

Dr. Begley's report occupies about three pages. Mr. Sankey, the medical superintendent of the female department, gives us some interesting details connected with his department. The two cases of accidental injury to patients, mentioned by Dr. Begley, are of interest, and should be read. In speaking of treatment, Mr. Sankey remarks:—

"The first and chief aim is to obtain for the patient rest for the affected organ—mental inactivity or mental rest. This is the object of what is called the moral treatment; a rest of mind is not to be obtained by indolence, which is probably more irritating than soothing, but by light amusements, by diversion. The means of amusement have been accorded freely during the past year: the chief of which have been walks in the neighbouring country, a summer entertainment in the open field, a weekly dance during the winter months. Some of the patients have been supplied with hoops and skipping ropes; two patients were taken to the Crystal Palace, and a small party spent a day at Hampton Court. The effect of these means is greater than one could have imagined. One patient discharged during the past year, told me, sometime after her recovery, that the first thing she could remember on the return to her reason, was crossing the Thames in a boat on going on one of these excursions to Kew. This patient was suicidal, and obstinately refused food; it was on this excursion that this propensity left her and did not again return."

The chaplain's report contains matter worthy of the most careful consideration. His position is one of delicacy and difficulty, requiring much judgment to guide him in the proper performance of his sacred and solemn duties. He says:—

"In those cases in which there is really or even only apparently a *religious element*, I believe the following points to be generally observable: first, that a total disregard of the obligations of religion is, in insanity, often followed by dæmonomania, or else by a species of wild fanaticism, the evidence or effect of madness, but certainly not the cause—secondly, that a merely formal service, in which the heart has no part, fails to give support in the time of trial, and is the precursor in madness of suicidal despondency—thirdly, that the flighty views which spring fpom imperfect instruction in the truths and requirements of the Christian religion, lay open the mind to the reception of the dogmas of ignorant enthusiasts, who mistake excitement for the motions of God's Holy Spirit within them, and find their representatives in our wards among the maniacal and melancholic cases; in these persons, who have mistaken excited feelings for signs of the favour of God, the depression which at length follows is regarded as an evidence that they are forsaken by the Almighty; life itself is insupportable to them, as they imagine that each moment increases the amount of their guilt, and while declaring their terror of death they seize the first opportunity of self-destruction: and fourthly, that the cases of those who have been brought up in certain religious views, and have subsequently from circumstances or from curiosity gone to places of worship where doctrines of an opposite kind have been taught, supply by far the largest amount of, so termed, religious insanity, when there has been at all a sincere, however erroneous attention, paid to spiritual concerns. Thus one brought up as an Arminian (W. P.) who had been taken to a chapel where high Calvinistic tenets were propounded, was alarmed, and on becoming insane thought himself excluded from mercy because not one of the elect. On the other hand, a patient, (C. W.) who had been brought up a Baptist, having been taken to a Wesleyan chapel, was excited by expressions which the ordinary frequenters of tliat place of worship would have scarcely noticed, and having become insane, is constantly harping upon her having undergone the new birth," &c.

Whilst the Rev. Mr. May asserts that deficient, defective, and unsettled views of religion are often more or less the *forerunners* of insanity, it is gratifying to hear him state, as the result of his observation, that sound and Scriptural religion, not merely *does* not *cause*, but tends to *avert* insanity, often sustaining the mind, which would otherwise have given way, and when a person has from other causes become insane, has still in very many cases afforded consolation, and tended, by calming the spirit, to aid in the recovery.

Mr. May remarks:—

"I do not regard it as a matter of importance to inquire whether the ministrations of the clergyman tend directly to the recovery of the patients."

Why should he not make such inquiries? Surely he would not be exceeding his legitimate duties by ascertaining what assistance he has rendered in his religious ministrations towards the cure of those who have been under his spiritual care.

In certain stages of insanity, when the medical treatment advised and carried into effect by the medical officers has properly fitted the mind of the patient for the judicious instruction of the chaplain, he may undoubtedly aid in promoting the recovery of the patient, by gently and discreetly bringing to bear upon the patient's mind the comforting, soothing, and holy influences of religion. Surely he might, with great propriety, inquire of the medical officers whether "his ministrations had tended indirectly to the recovery of the patients," and thus throw light upon an important point in the moral treatment of the insane.

Mr. May is evidently anxious not in the slightest degree to interfere with the legitimate functions of the medical officer, and we commend him for his wisdom and good feeling. He says:—

"I urge those who are restored, to thank God who has blessed the remedies of the physician, and it is always a point with me to tell those whom I visit, that I come to them as the minister of the gospel, to teach them the things which belong to their souls' peace and comfort, and that with their being in the asylum I have nothing to do. When this matter is settled, it rarely occurs that I am unable to obtain a quiet hearing even from those most anxious to leave, or most distressed in mind, and if, after a few words of kindness and instruction and a short prayer, I have left, as very frequently I have, a patient sitting quietly in the ward, reading a book, or, in the case of a female, employed with a simple piece of work, I consider that at least an opening has been afforded for the more efficient application of the remedial measures, which belong to the province of the physician.

"My intercourse," says Mr. May, "with the insane, convinces me that it is our duty to act with respect to the immortal soul, as if no lesion of the brain existed. The action of the mind upon the material organ, and the reaction of the diseased brain upon the mind, are matters which can never be explained satisfactorily—for *sound philosophy takes no cognizance of such a connexion.*"

We are not disposed to criticise too captiously the phraseology of this passage. We feel, however, assured from the context that Mr. May does not really mean what is implied in the preceding quotation. Mr. May asserts that it is the clergyman's "duty to act with respect to the immortal soul, *as if no lesion of the brain existed.*" This is manifestly erroneous. *It is* the duty of the chaplain, in his ministration, to consider with great care the existing lesion of the brain, for his attempt to influence religiously the mind of the insane might, in certain morbid conditions, or, to use Mr. May's own term, "lesions" of the brain, be productive of the most dire and disastrous consequences.

Again, we ask, does not "sound philosophy take cognizance" of the connexion between "the action of the mind upon the material organ," and "the reaction of the diseased brain upon the mind?" The practical psychologist may be unable to explain satisfactorily the nature of their mysterious connexion, but he certainly fully recognises the fact, and "takes cognizance of the connexion." Having thus cursorily glanced at Mr. May's interesting record, we next, in rotation, come to the "Matron's Report." With what a glorious exordium does Mrs. Macfie open her matronly battery! How proud, how gratified, must she have felt; what an intense consciousness this model of matrons must have had of her noble and elevated position, when, with her "grey *goose* quill" (we have no wish to perpetrate an odious pun) she sat in profound contemplation before penning the sublime and heroic passage with which she commences her report: —ANNO DOMINI 1856 HAS PASSED PEACEFULLY OVER THE FEMALE SIDE OF THE ASYLUM."

At the Colney Hatch Asylum 137 male patients were admitted during the last year, of which—

"The types and complications of the disease, were as follow:—

| | |
|---|---|
| Mania, uncomplicated | 18 |
| Melancolia " | 18 |
| Monomania " | 25 |
| Dementia " | 14 |
| Mania complicated, with General Paralysis | 4 |
| " " " Epilepsy | 2 |
| Dementia " " General Paralysis | 31 |
| " " " Epilepsy | 20 |
| Congenital Idiocy and Imbecility | 5 |
| Total | 137" |

Contrasted with this statement, it may be mentioned that—

| | |
|---|---|
| There have been discharged, Cured | 38 |
| " Relieved | 14 |
| " Unrelieved | 2 |
| " Died | 76 |
| Total discharged and dead | 130 |

Respecting the fatal cases, it is interesting to observe—

"A large number of the deaths has been ascribable to disease of the brain in association with 'general paralysis,' no less a proportion than 57 per cent. having been thus occasioned. Indeed, the number of patients who have succumbed to this exhausting form of disease, during the past year, is almost equal to that of the average of residents so affected; yet their number does not diminish, thirty-five new cases having been received, and ever and anon the case of some older resident becoming thus unfavourably and fatally complicated.

| | |
|---|---|
| Of this form of paralytic disease, have died | 44 |
| Of epilepsy | 14 |
| Of phthisis | 6 |
| Of atrophy, and decay of age | 9 |
| Of suicide | 1 |
| Total | 76" |

In the female department of the Asylum—

"The admissions for this year have amounted to 140 in the female department of this institution, the discharges 40; of these, 24 were recovered, 10 relieved, 3 removed to a workhouse, 3 to other asylums, and 4 upon trial; whilst 61 have died during the past year."

Again, with reference to the mortality, which amounted to 61 cases, the ratio was—

"Rather more than eight per cent., nearly double that of last year; a circumstance not altogether unlooked for, considering the amount of extreme chronic disease that is prevalent among so large a class of patients, many of whom have been in confinement for several years prior to their admission into this institution. The causes of death have been the following, viz. :—

| | |
|---|---|
| Phthisis | 14 |
| „ with epilepsy | 4 |
| Bronchitis | 1 |
| Broncho-pneumonia | 4 |
| Hydrothorax | 1 |
| Disease of the heart, dilatation | 1 |
| Gangrene of feet from disease of heart | 1 |
| Epilepsy | 6 |
| Paralysis, convulsions and coma | 16 |
| Chronic gastritis | 1 |
| „ peritonitis | 1 |
| Atrophy | 1 |
| Scrofula | 1 |
| Cancer of mouth, tongue and fauces | 1 |
| Suicide | 1 |
| Typhoid fever | 1 |
| Natural decay | 6 |

"Thus it appears that rather more than one third died from disease of the chest and respiratory organs, which seems to be one of the most fatal concomitants to mental disease, next to paralysis and epilepsy."

At the Surrey County Asylum, in the male department,—

"The discharges and deaths have been as follows :—

| | |
|---|---|
| Recovered | 61 |
| Uncured | 28 |
| Died | 75 |
| Leaving in the Asylum | 407" |

Again, by the Report it appears—

"The deaths have resulted from the following causes :—

| | |
|---|---|
| General paralysis | 22 |
| Apoplexy | 3 |
| Epilepsy | 4 |
| Pulmonary disease | 14 |
| Disease of the heart | 2 |
| Decay of nature | 7 |
| Exhaustion and other causes | 23 |

"The mortality is rather higher than usual, owing chiefly to a large number of cases of general paralysis, which had accumulated, and terminated fatally; and also to the fact (I regret again to report to you) that several patients when admitted were in so advanced a stage of bodily disease, that they died within a few weeks. I think the removal of such cases to an asylum is an evil, which ought not to be sanctioned by relieving officers and others, whose duty it is to sign the certificates of admission."

On the female side of the same institution,—

"During the year 92 have been admitted, 37 have been discharged recovered, 21 have been removed by their friends or respective parishes, and 36 have died; leaving in the asylum this day 515 female patients.

"One Coroner's inquest has been held, a verdict being returned of 'Natural death from an epileptic fit.'

"The causes of death have been as follows, viz.:—

| | |
|---|---|
| Paralysis | 10 |
| Epilepsy | 6 |
| Cerebral disease | 3 |
| Decay of nature | 7 |
| Pulmonary disease | 4 |
| Hydrothorax | 1 |
| Erysipelas | 1 |
| Disease of the heart | 1 |
| Fever | 2 |
| Scrofula | 1 |
| Total | 36" |

Much has recently been said in reference to the use of prolonged shower baths at this Asylum. Into that subject it is unnecessary again to enter. We, therefore, shall only quote the new regulations which the Committee have recently promulgated:—

"First. That neither of their medical officers shall in future administer any shower-bath without entering in the 'case book,' and reporting to the committee at their next meeting, the cause, the duration, and the effect thereof, together with the name of the patient on whom, and the date when it was applied.

"Secondly. That inasmuch as such baths may be dangerous in cases in which the heart of the patient is diseased, the medical officer regard it as his duty in all cases, before such a bath is administered, to examine the patient very carefully, to ascertain, as far as may be possible, whether by reason of diseased heart or other disease such a bath may not be dangerous.

"Thirdly. That for the future no new treatment of the patients, or extension of the previous practice, which may by possibility lead to results dangerous to the life or injurious to the health of the patients be left to the attendants; but that the effect of such new treatment, or extension of previous practice, be carefully watched by the medical officer who prescribes it, and fully reported in the 'case book.' "

At the Devon County Asylum, the last Report issued states:—

"During the past year 156 patients have been admitted, of whom 85 are men and 71 are women. The number of patients at the commencement of the year was 478. The average number resident has been 490; and the number resident at the present date is 520, of whom 232 are men, and 288 are women. Forty patients have died, of whom 21 were men, and 19 were women. Seventy-two patients have been discharged, of whom 28 were men, and 44 were women. The mortality has been in the ratio of 8 per cent. to the average number resident, and in that of 6·5 per cent. on the total number under treatment. Of the 72 patients discharged, 65 were recovered, 3 were discharged as relieved, and 4 are absent on trial.

"The admissions during the past year have been remarkable for the number of patients with propensities to commit suicide, and for the urgency of the symptoms displayed by them. No fewer than 55 of the patients admitted were stated on their admission papers to suffer from this lamentable propensity. Some were admitted with throats actually cut, and others with marks of violence inflicted for the purpose of self-destruction."

The Dorset County Asylum Report next presents itself to our notice. According to this official document,—

"On January 1st, 1855, there were in the asylum 148 patients—viz., 66 males and 82 females, since which time 26 males and 38 females have been admitted, 18 males and 20 females discharged, and 4 males and 11 females have died, and removed to Fisherton Asylum 3 males and 6 females. The number on the books now is 67 males and 83 females, and at Fisherton 21 males and 26 females; therefore the whole number belonging to the county and actually under treatment is 197—viz., 88 males and 109 females.

"It will be observed that the number admitted this year is the largest ever recorded since the opening of the Asylum. In the year 1846 the admissions were 27 males and 29 females—total, 56; whilst this year the numbers were 26 males and 38 females—total, 64."

The Lancaster County Asylums comprise two institutions, viz., one at Lancaster and another at Ramhill. Respecting the first-named, it appears,—

"During the past year 186 patients have been admitted—men, 95; women, 91—being 2 more upon the whole than were received in the previous year. Although the character of these cases does not differ materially from the admission of former years, nor call for any special notice, yet it is worthy of remark that in asylums where no selection of patients can be made, it must of necessity happen that the yearly influx of epileptics and patients suffering from general paralysis, or in other words from incurable forms of disease, must add greatly to the permanent residents. This is an evil incident to all county asylums, and the older the establishment the more oppressive is the burden."

Again, at Ramhill, it appears,—

"Of the 92 patients admitted during the year, 19 had had former attacks of insanity, and 12 had previously been under treatment in asylums; 6 at this institution, 2 at the Lancaster Asylum, and the remaining 4 at other public asylums. Sixty-one patients—namely, 28 men, and 33 women, have been discharged recovered, during the past year. Of these, 6 men, and 5 women, were previously out on trial for a month, and one man for two months. The recoveries during the year have been at the rate of 66·30 per cent. upon the admissions, a much higher proportion than has occurred in any previous year since the opening of the asylum. This high per-centage is in some measure due to the recovery of several patients whose mental malady had long assumed all the characters of chronic insanity."

Numerous other reports—some of much value—well deserve being specially named in the present analysis of important documents of that description, with which our table is now covered; all being kindly forwarded by the respective medical superintendents or other official gentlemen connected with the different establishments described; to all of whom our best thanks are justly due, and are now expressed. We should have liked to notice each at considerable length—commensurate with their intrinsic importance—but our limited space now at command renders that task, however agreeable it would unquestionably prove, at present out of the question. Such being the position in which we are thus placed, and against whose imperious necessity it is difficult to contend, we can only now briefly allude to the important public documents still remaining for analysation.

Mr. Green, medical superintendent of the Birmingham Lunatic Asylum, in his last report, states:—

"During the year, 126 patients have been admitted, of whom 78 were males and 48 females; which together with the 285 left at the end of the previous year, make a total of 411 who have been under treatment. Of this number, 57—being 45 per cent. upon the admissions—have been discharged cured, and 11 others so far improved as to be fit for restoration to their homes. Thirty-three of the admissions were private patients, all of whom were males, a circumstance which will be again adverted to in a future part of the report."

Regarding the Essex Asylum, Dr. Campbell, the medical superintendent, observes:—

"Between the 25th of December, 1854, and the 25th of December last, there were received into the asylum, 61 male and 68 female patients; total, 129. There died within it, 20 male and 28 female patients; total, 48. And there were discharged from it, unimproved, 2 males; improved, 2 males; and recovered, 22 males and 28 females; total recovered, 50. Since it was opened in September, 1853, up to the 25th December last, there have been received into it, males 265, females 303; total, 568. On the 25th December last there were remaining in it, males 139, females 195; total, 334."

Dr. Huxley, of the Kent Asylum, remarks in his report, that the number of patients admitted during the past year was 192, of whom 108 were male, and only 84 female lunatics; 71 recovered, and 61 died, both sexes comprised. The aggregate inmates remaining being 599, on the 4th of July last.

At the Manchester Royal Lunatic Hospital, Dr. Dickson reports:—

"During the twelve months which have since elapsed, forty-five cases have been admitted, nineteen of which are males and twenty-six females; making altogether one hundred and thirteen patients who have been under treatment during the year. Of this number forty have been discharged, leaving at this date seventy-three patients in the hospital. The average number daily resident throughout the year has been 75·7. Of the forty patients discharged twenty-two were cured, being in the ratio of nearly 49 per cent. on the number admitted, and 29·25 per cent. on the average number resident."

From the Leicestershire and Rutland Asylum, of which Dr. Shaw is the visiting physician, it appears:—

"The greatest number of patients in any one day was 310, the number in the house on the 31st December, 1856, was 304, being an increase of 9 on the number present on the 31st December, 1855.

"During the past year 107 patients were admitted; the number under treatment during the year was 402, of these 53 have been discharged cured, viz. 23 males and 30 females, and 11 have been discharged relieved, viz. 7 males and 4 females, making a total of 64 who have left the asylum.

"The number of deaths during the year has been 29, and the rate of mortality on the average number of patients has been 9·60 per cent., as will be seen by reference to Table No. 2,—a rate of mortality which, though slightly above that of last year, is still below the average rate, during the existence of the asylum."

In the Lincolnshire Lunatic Asylum, at Bracebridge, whereof Dr. Palmer is medical superintendent:—

"It will be observed, that at the close of the year 1854 there were 243 patients in the asylum, of whom 120 were men, and 123 women, and that during the year 1855, 32 men and 30 women were admitted; thus making the total number under treatment 305, viz.—152 men, and 153 women.

"Of these, 2 men and 2 women were discharged relieved, 13 men and 10 women were sent out recovered, and 10 men and 13 women died, making the total of discharges and deaths 50; viz., 25 men and 25 women, and leaving in the asylum at the end of the year 255 patients, consisting of 127 men and 128 women.

"The average daily resident numbers were 122·15 of men, and 127·37 of women, being 249·52 of both sexes collectively."

According to the report of Dr. Allen, medical superintendent of the asylum at Abergavenny :—

"At the close of 1855 there remained in the asylum 257 patients, viz., 114 males and 143 females. During the year 1856, 116 patients have been admitted, viz., 62 males and 54 females: of these, one male was a criminal lunatic, admitted under a warrant of the Secretary of State.

"The re-admissions during the year amounted to 17, viz., 7 males and 10 females.

"The discharges during the year have amounted to 57, viz., 34 males and 23 females: of these, 33 males and 20 females were recovered or relieved; 1 male (the criminal lunatic) succeeded in effecting his escape, and 3 females were discharged on the solicitation of friends, and on the agreement that they were to be properly looked after and taken care of.

"The deaths during the year have amounted to 34, viz., 20 males and 14 females; 15 of the deaths occurred in persons advanced in life, varying from 58 to 83 years of age; and the average duration of life in the whole number was 51 years."

Dr. Nesbitt, superintendent of the Northampton General Asylum, says, in his recent report, that :—

"During the past year we have received 64 inmates, 26 being private and 38 pauper. This is a smaller number than we have been accustomed to admit for the last few years; but it will be recollected that we not only served notices in 1854 on the different Unions in the County that the house was then more than full, but that we came to the resolution of not admitting a private patient under one guinea a week.

"Of recoveries in the last year there have been 21. Many of these expressed both in prose and verse their thankfulness that in the hour of their extremity they here found 'a city of refuge.' Most of them are now engaged in their different vocations, whilst others are able to lead a quiet life among their friends.

"The mortality is large in proportion to the numbers in the house. It amounts to 34, on the average daily number of 261, or, in other words, something over 13 per cent. The deaths in 1854 were only 10½ per cent. The average age at death for the past year was 48. Of those admitted during the year 10 died, all being at the time of admission in a more or less enfeebled state. One died within a few hours of arrival, having been previously suffering from severe depletion, and the fatigue of a long journey. It is a remarkable fact that the deaths should be in an inverse proportion to what might be expected; for while out of the 38 pauper patients 3 only died, as many as 7 died out of the 26 private patients. The explanation, however, of this apparent anomaly is not difficult. For there are greater facilities in the classes above pauperism to meet the infirmities of their friends at home, and better means of protecting them there, than the poor can command. Hence their condition, on admission, is often, both physically and mentally, a more deteriorated one. Many of them have already been inmates of private asylums, and are sent here when their cases are hopeless, and their habits depraved and offensive."

From the General Asylum, near Nottingham, Dr. Williams, the visiting physician, and Mr. Stiff, the superintendent, say:—

"They have had greatly increased admissions in comparison with the last year, many of them of a dangerous and most suicidal character.

"The numbers admitted have been 66, viz., 36 men and 30 women. The great majority of these were from Nottingham and its immediate neighbourhood.

"Of the 66 admissions, one half, viz., 15 females and 18 males were suicidal. Their unhappy state was, in many instances, produced by distress of mind from losses in business, absence of work, and consequent poverty, which resulted from their occupations and employments in the several branches of trade in and near Nottingham.

"There has been an excess of male admissions in both classes of patients, notwithstanding that the excess of the female population in the town of Nottingham is 4277, and in the county 903. We believe this excess in proportion of admissions to have been accidental, as sometimes the one sex has predominated in the Asylum and sometimes the other.

"Sixteen patients had hereditary disposition to insanity, and 9 had the disease induced by habits of intemperance.

"The number of discharges and deaths were 71; of these we have discharged cured, 23; relieved, 9; harmless chronics, 11; not improved, 4; died, 24. The deaths were chiefly from the usual causes which prevail in asylums, viz., apoplexy, paralysis, epilepsy, and phthisis pulmonalis."

At the County and Borough Asylum of Snenton, it is stated by the Report of Dr. Stiff, the resident physician, that:—

"On the 31st December, 1855, there remained 230 patients,—viz., 120 men and 110 women. During the year 1856, 45 men and 44 women were admitted, together 89, making a total of 319 under care and treatment. The recoveries have been 30, in equal proportions of the sexes. Ten were discharged relieved, 3 not improved, 17 died, and 36 were removed by the Union authorities as harmless chronic lunatics. There now remain 120 men and 103 women.—Total 223."

By the report of Dr. Oliver, medical superintendent of the asylum for the counties of Salop and Montgomery, it appears that:—

"On the 1st of January, 1856, the number of patients in the asylum was 315 (viz., 149 males and 166 females). In the course of the year exactly 100 patients (viz., 52 men and* 48 women) were admitted; 37 (viz., 17 men and 20 women) were discharged recovered; 18 (viz., 9 men and 9 women) were discharged relieved; 3 (viz., 1 man and 2 women) were discharged not improved; and 33 (viz., 19 men and 14 women) died.—The number of patients remaining in the asylum on the 31st December, 1856, was consequently 324 (viz., 155 men and 169 women) an increase on the number of those remaining at the end of the previous year of 9 (viz., of 6 men and 3 women)."

Dr. Boyd, the superintendent of the Somerset County Asylum, observes, in the ninth report of that institution, that:—

"During the year 1856 there were 133 admissions, 73 males and 60 females. 30 males and 35 females were discharged recovered; 4 males and 6 females relieved; 10 males and 1 female not improved; 1 male and 2 females out on probation; 16 males and 20 females died; remaining 167 males and 191 females. The number of males discharged not improved is more than usual, four male patients from Bedminster having been twice discharged during the year; first in February, in consequence of the higher rate of payment charged to boroughs which had not contributed to the building having been refused; these patients were, however, again returned in a few days, and the higher rate has since been paid: on the 27th December they were finally transferred to the Hospital for Lunatics for the borough of Bristol. Although there is an increase of 9 remaining, over the corresponding period of the previous year, still it is hoped that no further addition to the asylum will be required."

At the Staffordshire County Asylum, Dr. Bower says:—

"The admission of patients into this asylum from January 1st to December 31st, 1856, has very nearly corresponded to that of the preceding year; the numbers being in 1855—174; in 1856—172.

"The average of those resident in 1856 was 412, as compared to 398 in 1855.

"410 patients remained in the house on December 31st, 1856; whilst 406 was the amount at the end of 1855.

"The average number of recoveries in the two years has been nearly equal, since on a mean resident total of 412 in 1856, there were 59·39 per cent.; whilst in 1855, the average being 398, the recoveries were 52·29; thus showing a slight advantage for 1856 over the former year."

Dr. Kirkman, in his late report of the Suffolk Asylum, states that during 1856 the admissions of new patients amounted to 88, comprising 42 males and 46 females; 20 males, and 18 females having left cured, while 15 of the former, and 12 of the latter sex died, or a total of 27 deaths; while the aggregate

* None were both admitted for the first time, and re-admitted within the year, excepting one woman. This circumstance reduces the number of females received to 47 individuals, and the aggregate number of individuals of both sexes admitted during the year 1856 from 100 to 99.

number remaining on the 31st of December, were 130 males and 155 females, or 285 patients altogether.

According to the sixth annual report of Dr. Thurnam, medical superintendent of the Wilts County Asylum :—

"At the commencement of 1856 there were 301 patients under care—namely, 141 men and 160 women. During the year, 100 have been admitted—39 males and 61 females; of whom 20—6 men and 14 women—were re-admissions. The number in the asylum at the date of this report, is 314—145 men and 169 women. The average number resident during the year has been 304.

"Of those discharged, 52 were registered as recovered;—a proportion exceeding half the number of the admissions, and which cannot be regarded as other than favourable.

"No serious epidemic disorder of any kind has prevailed; and the general health of the patients has been good.

"There have been 28 deaths—16 of men and 12 of women; the mortality being at the rate of 9·21 per cent., which is 2 per cent. lower than the mean rate of mortality during the 5·35 years since the opening of the asylum.

| Mean Annual Mortality. | Males. | Females. | Both Sexes. |
|---|---|---|---|
| For the year 1856 . . . | 11·53 | 7·25 | 9·21 |
| For 5·35 years*—1851-56 . | 13·15 | 9·46 | 11·14 |

"Among the deaths, have been four from general paralysis—a disease so fatal to the insane, and almost peculiar to them. The number of deaths from this disease now recorded is greater than in any former year. In the case of one epileptic patient found dead in bed, a coroner's inquest was held; the verdict being, 'died suddenly by the visitation of God.' Death in this instance probably occurred from a fit of epilepsy during sleep, which at once proved fatal; the patient appearing to have died almost without a struggle, and being found in a recumbent attitude, and with an aspect of perfect composure."

In the report of the asylum for the county and city of Worcester, Dr, Sherlock, the medical superintendent, says :—

"At the end of the year 1854 there remained under treatment in the asylum 218 patients, 104 males and 114 females. During the current year 101 patients have been admitted, 53 males and 48 females, making a total of 319 which have been under treatment: 38 patients have been discharged recovered, in equal numbers from either sex. Twelve have been removed improved, 7 males and 5 females, not a few of whom were at the period of their removal in an advanced stage of convalescence, while others were cases of a chronic and, so far as could be judged, of a harmless character, for whose care and protection various provision would be made by friends or others. In each case of this description particular regard has been paid to the position in which the lunatic was to be placed. This number also includes the cases of patients transferred to other asylums, subsequently to the fixing of their settlements.

* Viz., from the opening of the Asylum, Sept. 19th, 1851, to the end of the year 1856.

"The deaths during the year amounted to 39, of whom 24 were males and 15 females.

"The average number resident throughout the year was 231, 110 males and 121 females.

"The number of cases admitted and under treatment exceed those recorded in the Report of the previous year by 13 and 20 respectively."

At the pauper asylum for the West Riding of York, Dr. Alderson, the medical superintendent, reports that:—

"On the 1st of January, 1856, the number of patients resident in the West Riding Asylum were 356 males and 411 females; since that time 141 males and 155 females have been admitted, making a total under treatment of 1063, and an average daily number resident throughout the year of 803.

"The discharges during this period were 279 from all causes, viz., 55 males and 67 females recovered; 23 males and 29 females relieved; 1 male not improved; and 50 males, 54 females died; thus leaving 784 patients resident on the 31st of December."

Dr. Cassow, of the Hull Borough Asylum, reports:—

"The admissions to have been equal to the discharges and deaths combined, viz., 15 males and 17 females, so that precisely the same number of each sex were in the asylum on the first day of the year as the last, a somewhat remarkable circumstance; not more so, however, than that anomaly which the statistics of the asylum present in the relative proportion of the sexes.

"Since the opening of the asylum, the males, with one or two very short exceptions, have preponderated over the females, to the extent of from 4 to 6, whereas, taking the asylums generally throughout the country, probably nearly all contain a larger proportion of the softer sex; the female being apparently more prone to insanity than the male.

"The percentage of recoveries on the number admitted, was $56\frac{1}{5}$, in addition to which 4 were discharged much relieved, of whom 3 continued so well after their release as to be convalescent up to the present time; therefore the average number of cures were, in fact, nearly 66 per cent., being higher than that of the English asylums generally.

"The percentage of deaths, taken on the mean daily number resident ($89\frac{2}{3}$) was $11\frac{1}{5}$, a shade lower than that of the previous year, and I believe 2 or 3 below the average rate of the English institutions."

At the North and East Riding Asylum, Clifton, Yorkshire, Dr. Hill, the superintendent, reports that:—

"During 1856 the admissions were 95, of whom 58 were male and 37 female lunatics; 11 males and 9 females were discharged cured, while 16 of each sex died, making 32 deaths altogether. The total inmates remaining under treatment, on the 31st December last, being 191 male and 164 female lunatics; thus making an aggregate of 355 individuals then resident."

The report of the medical officers of the Norfolk Lunatic

Asylum, at Thorpe, must also be noticed. According to this public document, it is stated that :—

"On 31st December, 1855, there were in the institution 293 inmates, namely, 135 male and 158 female patients. During the past year 85 inmates have been admitted, namely, 41 male and 44 female patients. The whole number under our care has therefore been 378—175 male and 202 female patients. The number of discharges has been 41, namely, 21 male and 20 female patients; of these 39—20 male and 19 female patients have recovered, and 2—1 male and 1 female were relieved. During 1856 no patient has been removed without improvement. The number of deaths in 1856 has been 37—18 male and 19 female patients."

Before taking leave, for the present, of the medical superintendents of English asylums, and the very valuable professional Reports which they annually lay before their respective Boards of visiting justices, or other public functionaries, one more of these often instructive documents yet remains to be mentioned, viz., that of Dr. Bucknill, the experienced medical superintendent of the Devon County Asylum, who says :—

"During the past year 156 patients have been admitted, of whom 85 are men and 71 are women.

"The number of patients at the commencement of the year was 478. The average number resident has been 490: and the number resident at the present date is 520, of whom 232 are men and 288 are women.

"Forty patients have died, of whom 21 were men and 19 were women.

"Seventy-two patients have been discharged, of whom 28 were men and 44 were women.

"The mortality has been in the ratio of 8 per cent. to the average number resident, and in that of 6·5 per cent. on the total number under treatment.

"Of the 72 patients discharged, 65 were recovered, 3 were discharged as relieved, and 4 are absent on trial.

"The admissions during the past year have been remarkable for the number of patients with propensities to commit suicide; and for the urgency of the symptoms displayed by them. No fewer than 55 of the patients admitted were stated on their admission papers to suffer from this lamentable propensity. Some were admitted with throats actually cut, and others with marks of violence inflicted for the purpose of self-destruction. The form of disease under which the greater part of these patients laboured was that of melancholia: in a considerable number, however, the symptoms were those of high cerebral excitement; and, in the absence of the suicidal propensity, would have been considered as undoubted cases of mania. The presence of this symptom would not seem to afford a sufficient reason for excluding them from a class to which they would otherwise belong. The manifestations of insanity observed in the wards of this institution as fully justifies the use of the term 'suicidal mania,' as that of 'suicidal melancholia,' which has long been in common use.

"In suicidal mania the head is usually hot, the aspect fierce, and the general symptoms those of excitement: the propensity to self-destruction is frequently accompanied by a general tendency to commit acts of violence. The general excitement also undergoes marked periods of remission, and during these periods the suicidal passion suffers abatement. These cases indeed, in addition to all the ordinary symptoms of mania, present a violent and impulsive desire to commit self-destruction:—this desire, or passion, is too urgent and vehement to be correctly designated by the common term—inclination or propensity. It is sometimes associated with delusions of such a nature that they may be supposed to have given rise to the morbid desire, or to have been occasioned by it; or, what is yet more probable, to have originated in the same morbid condition of the brain. Thus one man believed that he saw demons around him, who called upon him to join them in an incarnate state: another believed that he heard a voice from heaven, calling upon him to sacrifice himself in order to put his enemies to shame. More usually, however, in suicidal mania there is no delusion bearing upon the morbid passion."

Other important topics discussed by the able author just quoted might be advantageously noticed in the present analysis; but however anxious to extend our review of this, and likewise of various previously-cited official reports, we must refrain reluctantly, in order to proceed towards another portion of the British Empire—namely, North Britain.

Although the condition of lunatics in Scotland has formed the subject of a special article in a former part of this number, when speaking of the Report issued by Parliament, and drawn up by the Lunacy Commissioners sent to investigate the present state of lunacy in that portion of Great Britain, nevertheless, several annual reports detailing the transactions which have characterized the past year at some of the chartered asylums in that country, deserve perusal, especially as they amply show that the barbarities which have been unveiled at other places, did not in any manner prevail within the precincts of those useful public institutions. That is a truth which cannot be too extensively disseminated throughout the Empire.

The first public receptacle for lunatics to which attention will now be directed, is the Glasgow Royal Asylum.

There, during 1856, the admissions were greater than in the preceding year, the total number being 217, of whom 118 were males, and 99 females, thus showing the former sex predominated. Respecting the patients admitted:—

"Several of all classes when received were hopelessly ill, their malady being complicated with epilepsy, paralysis, or some other organic disease tending to shorten life. One female had had her arms so long bound with cords before she was brought hither, that she had lost the power of one of them completely; another had a miscarriage shortly after her arrival; while a third had been seventeen years insane before admission, having been kept at home during the whole of that period."

In reference to the causes usually assigned :—

"It is generally found that, of the insane, the unmarried are in excess of the married and widowed. Here the opposite has sometimes prevailed. This year the two classes are nearly equal.

"When the hereditary cases, and those who had previously laboured under insanity are excluded, it will be found that among the males the cases arising from physical causes greatly predominate over those arising from moral, while among the females they are nearly equal.

"In two females the disease arose from immersion in hot baths at too high a temperature. In one case, a plethoric young woman, the temperature of the water was 111° Fahrenheit. She became maniacal a few hours after being taken out. This patient had previously laboured under insanity, but had been long well before she was subjected to a trial of this powerful excitant. The other patient had suffered occasionally from epileptic vertigo, for eighteen months or so, when she was placed in a bath at a temperature of 110°, and became insane shortly afterwards. Although the cases arising from intemperance are fewer than last year, we still find that it occupies the first place among the distinctly ascertained causes. The numbers under this head, however, do not give a fair view of the actual number of individuals admitted from that cause, as two were admitted and dismissed more than once from intemperance during the year. Those addicted to this vice are in general easily cured of the first attack; but after repeated seizures they sink into confirmed and incurable insanity."

Respecting dismissions :—

"The aggregate numbers were rather fewer this year, but the number cured has been much higher—being this year 91, and last year 69. Our returns confirm the general belief that insanity is more curable in females than in males. This, however, is easily accounted for. Females lead a more quiet and regular life; they are much less exposed than males; they seldom become paralytic—which is the most unfavourable of all the complications; and their insanity is often dependent on the derangement of the functions peculiar to their sex.

"It is interesting to notice that a considerable proportion recovered at an advanced period of life. Thus, twelve males and nine females were cured who were upwards of fifty years of age; two, a male and a female, were nearly eighty. These cures were nearly equally distributed throughout the year. The greatest number of recoveries has, as usual, been among those who laboured under the maniacal form of the malady. The majority of those who recovered had been placed under treatment at an early stage of the disease. Thus, of the 91 cured, 64 had been admitted within two months of the commencement of their illness. A few were dismissed well after many years' residence."

Again:—

"The deaths were less in number this year than the last, although several patients were in a dying state on admission. Others had long suffered from organic chest affections. One of the male patients died twenty-two hours after admission, having been in a state of collapse when admitted. A female was brought in labouring under advanced phthisis pulmonalis, and survived only a few days. A number of the patients who died were of an advanced age. Some of them had been from six to twelve years in the house. The chief cause of death was phthisis pulmonalis. A male patient died from gangrene of the lungs. The extreme and peculiar fœtor of his breath, for some days before his death, indicated that in all probability this condition existed. By a *post mortem* examination, the upper half of the right lung was found to be a gangrenous mass."

The only other point of interest to which space allows reference to be now made, is, the pathological appearances observed in the cases which terminated fatally. According to the Report now under review, it is stated that

"A considerable number of bodies were inspected during the year. In every case the brain and its membranes were found to be more or less diseased. The following were the chief morbid conditions in the head:—Skull thickened, bones hard, compact without diploe; dura mater thickened, and in several instances considerable osseous spiculæ were found developed in it; pia mater opaque, thickened and infiltrated with serous fluid; serous fluid in the ventricles, with ulceration of their surface. Some brains, where chronic and violent mania had existed, were unusually hard. One well marked instance of softening of both grey and white matter of the brain was found: the patient had been acutely maniacal. In the great majority, disease was also found in other cavities, and was the immediate cause of death."

The treatment, occupations, and amusements of the patients are next adverted to by the physician superintendent, Dr. M'Intosh, but into those interesting questions we cannot now enter. They seem to have been much of the usual character, fully indicating that these important features in the management of all well-regulated public asylums were not overlooked at this large institution.

The Dundee Asylum next occupies attention. At this public institution

"During the past year 35 patients have been admitted to the Asylum, of whom 22 were males, and 13 females.

"At the date of the last Annual Court there were 210 patients resident; thus 245 individuals have been treated in the Institution during the year. The daily average number resident having been 216.

"35 have been removed during the year, of whom 19 were recovered,

5 not recovered, and 11 have died; leaving at the close of the year 210 inmates.

"The number discharged recovered was thus in the proportion of 54 per cent. to the admissions. The number of patients admitted since the opening of the Institution amounts to 1625; and of these 742 have recovered, being within a fraction of 46 per cent. If, however, the patients still remaining under treatment be deducted, the per centage is raised to $52\frac{1}{2}$. And it is obvious that this deduction must be made if we would render the calculation as accurate as possible, otherwise the quota of recoveries which we may happily expect to be drawn from the 210 sufferers still under our care is unrecognised, and is made actually to throw its weight into the scale of non-recoveries.

"The mortality during the year has been 5 per cent. upon the average number resident, or $4\frac{1}{2}$ upon the whole number under treatment during the year. The average annual mortality during the last six-and-twenty years has been 5·78 per cent. Thus showing that the number passing away from our community by death has been somewhat below the average of past years, although exactly the same number of deaths have occurred this year as during the previous year. The measure of physical health and longevity with which our community has been blessed will be more easily appreciated, if it be mentioned that the most experienced authorities have arrived at the opinion that in an asylum such as this, devoted to the treatment of both upper and lower classes of patients, a mortality which exceeds 9 or 10 per cent. is usually to be considered as unfavourable, and one which is less than 7 per cent. as highly favourable. Our average annual mortality having been 5 and a fraction per cent., as above stated, indicates that the conditions tending to maintain bodily vigour are uncommonly complete, affording matter for much satisfaction."

Respecting the mortality, it appears

"Eleven patients have died during the year, of whom 9 were males, and 2 females. The diseases to which they succumbed were principally of that intractable nature which afforded little room for hope that the impending danger could be averted. 4 males and 1 female died of marasmus, or the exhaustion of the vital powers induced apparently by protracted and agonising delusions. One of these male patients presented the only instance during the year of abstinence from food to so determined an extent as to require the use of the stomach-pump. Several instances occurred in which, under the influence of various delusions, there was a temporary repugnance to take food, but the difficulty was always overcome by some other expedient short of the means referred to. In one of these instances, a gentleman refused all food under the impression that it was the vehicle of poison. No persuasion could shake his conviction. Interference became necessary; but when the stomach-pump was produced, and preparations made for its use, he took food voluntarily.

"There were two deaths, both males, during the year, from pulmonary consumption.

"Two persons, a male and a female, died of that formidable complication named the general paralysis of the insane.

"Of the remaining two male patients who died during the year, one was cut off by chronic bronchitis. The other sunk from an attack of inflammation of the lungs, following upon a very severe and almost fatal fit of epilepsy. During the last six years his mind had been quite a blank; he never uttered a rational sentence; and the jargon which occasionally fell from his lips was broken and disjointed, as if he had great difficulty in articulation. His movements of both extremities were unsteady and ill-balanced. His whole nervous system seemed to have undergone a process of disorganization; nevertheless, it was a remarkable fact that the *post mortem* examination failed to reveal any departure from the ordinary structure of the nervous apparatus. The usual appearances of recent and extensive inflammation were found in the lungs, which explained his death; but the epilepsy, together with the total destruction of the mental powers, and the impairment of the volitional movements, left no impress behind them, appreciable either to microscopical or other means of detection, to explain the formidable malady exhibited during life."

Dr. Wingett, the Medical Superintendent, subsequently discusses the occupation, amusement, and instruction of the patients, which laudable efforts continue to give a good return in augmenting the sum of health and happiness of the insane population placed under his charge. To enter into any details respecting the special means employed is unnecessary, as they appear similar to what have been mentioned in previous reports, and are, on the whole, analogous to those often employed elsewhere; while with their beneficial working the profession and public seem already generally familiar.

We had intended to allude to other public Asylums in Scotland, especially to those at Dumfries and Aberdeen, both highly deserving of notice, but this instructive occupation must be deferred until some future opportunity. The institutions of Ireland must likewise be deferred. In conclusion, however, we now would only remark that the Reports from all incontestably manifest how essentially beneficial early treatment, with proper and safe surveillance, always prove, especially in an important class of lunatics—viz., who often become either dangerous to others or to themselves, not only under ordinary circumstances, but even where carefully watched, and having the many advantages possessed by every well-regulated public institution for the insane.

---

⁂ *A copious abstract of Foreign Psychological Literature will appear in our next Number.*

